# New trimester-specific reference intervals for clinical biochemical tests in Taiwanese pregnant women-cohort of TMICS

**DOI:** 10.1371/journal.pone.0243761

**Published:** 2020-12-14

**Authors:** Hui-Ming Chen, Fu-Chen Kuo, Chou-Cheng Chen, Chia-Fang Wu, Chien-Wen Sun, Mei-Lien Chen, Chia-Jung Hsieh, Shu-Li Wang, Ming-Tsang Wu

**Affiliations:** 1 Research Center for Environmental Medicine, Kaohsiung Medical University, Kaohsiung, Taiwan; 2 Department of Family Medicine and Occupational Medicine, Kaohsiung Chang Gung Memorial Hospital, Kaohsiung, Taiwan; 3 School of Medicine, College of Medicine, I-Shou University, Kaohsiung, Taiwan; 4 Department of Obstetrics & Gynecology, E-Da Hospital, Kaohsiung, Taiwan; 5 PhD Program in Environmental and Occupational Medicine, Kaohsiung Medical University, Kaohsiung, Taiwan; 6 National Environmental Health Research Center, National Institute of Environmental Health Sciences, National Health Research Institutes, Miaoli, Taiwan; 7 Institute of Environmental and Occupational Health Sciences, College of Medicine, National Yang Ming University, Taipei, Taiwan; 8 Department of Public Health, Tzu Chi University, Hualien, Taiwan; 9 School of Public Health, National Defense Medical Center, Taipei, Taiwan; 10 Department of Public Health, China Medical University, Taichung, Taiwan; 11 Department of Public Health, College of Health Sciences, Kaohsiung Medical University, Kaohsiung, Taiwan; 12 Department of Family Medicine, Kaohsiung Medical University Hospital, Kaohsiung Medical University, Kaohsiung, Taiwan; 13 Graduate Institute of Clinical Medicine, Kaohsiung Medical University, Kaohsiung, Taiwan; University of Mississippi Medical Center, UNITED STATES

## Abstract

**Background:**

Because there are no published biochemical reference intervals (RI) for pregnant Taiwanese women, we used an established islandwide birth cohort, the Taiwan Maternal and Infant Cohort Study, to establish RIs for important biochemical parameters in women during their 3rd trimester in Taiwan. Additionally, we compared the differences in these biochemical parameters between early third trimester (weeks 28 to 31) and late third trimester (weeks 37 to 40) of pregnant women as well as the differences in them between the third trimester and after delivery.

**Methods:**

Between 2012 and 2015, we recruited a total of 2,136 pregnant women from nine hospitals located in northern (n = 3), central (n = 3), southern (n = 2), and eastern Taiwan (n = 1) to receive regular prenatal health examinations during their third trimester (weeks 28 to 40). After exclusion, samples obtained from 993 eligible pregnant women were analyzed.

**Results:**

There were increases in both lower and upper normal limits for blood neutrophil, thyroid profile (triiodothyronine (T3) and thyroxine (T4)), testosterone, estradiol, and progesterone and decreases for RBC, hemoglobin (Hb), alanine aminotransferase (ALT) and creatinine (Cr) during their third trimesters. Women in their late third trimester (n = 378) had higher median RBC, Hb, aspartate aminotransferase (AST), Cr, thyroid-stimulating hormone (TSH), testosterone, estradiol, and progesterone and lower median platelet and insulin, compared with those in their early third trimester (n = 490). Twenty-three of the women had both third trimester and post-pregnancy data. After delivery, the women had lower median AST, ALT, insulin, T3, T4, testosterone, estradiol, and progesterone and higher median Cr, free T4, FSH, and luteinizing hormone (LH), compared to their third trimesters.

**Conclusions:**

Gestation-related changes in important biochemical parameters should be considered when evaluating clinical laboratory values in pregnant women.

## Introduction

Reference intervals (RI), which are upper and lower ranges of specific physiologic measurements, are often used as reference points when evaluating laboratory results for healthy people. They are often used to determine whether a patient needs further evaluation. Pregnancy induces several physiological changes in most organ systems, and along with these changes, come fluctuations in laboratory test values. Although most laboratories are well aware that pregnancy induces changes in normal laboratory values, they rarely provide healthy reference intervals for pregnant women. Without adequate RIs, there is increased risk of overlooking changes that might occur due to pathological conditions. There is also increased risk of misinterpreting normal changes as pathological.

Most tables of reference intervals for pregnant women focus on thyroid hormones [1–3], followed by blood routine [4, 5] as well as others including pregnancy associated plasma protein-A (PAPP-A), free β-human chorionic gonadotropin, tumor necrosis factor-α (TNF-α) and interferon gamma (INF-γ) [6, 7]. There are some sources that provide reference ranges or laboratory values for pregnant women in western countries [8–11] and there are several papers that provided them for pregnant women in some Asian populations [3–5, 12]. There are none in Taiwan. Using data obtained from Caucasian pregnant women to extrapolate to Taiwanese gravida requires caution since there may be ethnic differences. Therefore, we performed this study to (1) establish reference intervals for important biochemical parameters in pregnant women during their third trimester (weeks 28 to 40) in Taiwan, (2) compare the differences in these biochemical parameters between their early third trimester (weeks 28 to 31) and late third trimester (weeks 37 to 40), and (3) compare the changes in these parameters between the third trimester and after delivery. These reference values could potentially be used to help Taiwanese clinicians distinguish physiologic changes that occur during pregnancy from pathological ones.

## Materials and methods

### Subjects

Taiwan Maternal and Infant Cohort Study (TMICS) was established in October 2012, after the 2011 Taiwan phthalate-tainted food scandal [13]. The primary objective of this birth cohort study was to investigate environmental hazard exposure and the health status of mothers and children in Taiwan. The 2011 Taiwan food scandal started around April, 2011 and wound down in August 2011 [14]. Our previous study had found the estimated daily DEHP (di-(2-ethylhexyl) phthalate) intake in more than 99% pregnant women to be below the recommended tolerable daily intake (TDI) level (0.05 mg/kg/day) of European Food Safety Authority [13], suggesting the subjects in this study were not currently affected by the 2011 Taiwan food scandal.

**Fig 1:**
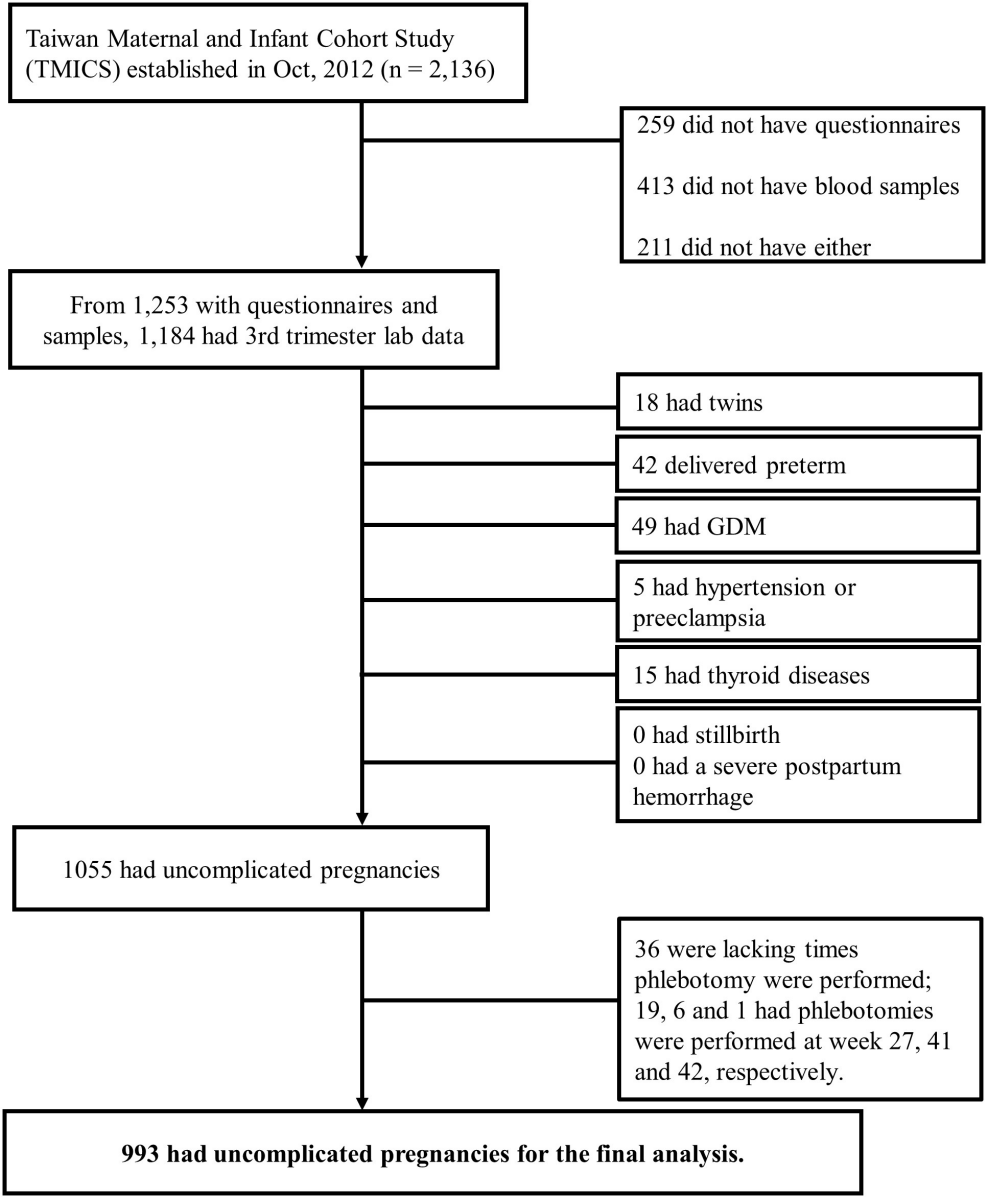
Study flowchart.

A total of 2,136 pregnant women were recruited from nine hospitals in northern (N = 3), central (N = 3), southern (N = 2), and eastern Taiwan (N = 1) between October 2012 and May 2015 to receive regular and routine prenatal health examinations, mostly between their third trimester and before delivery (Fig 1). Subjects belonging to this cohort were enrolled if they did not chronically use corticosteroids or immunosuppressant drugs, were 18- to 45-years old, and willing to participate. After excluding 883 women who did not answer questionnaires or provide blood samples, we were left 1,253 subjects. 1,184 of them had complete laboratory workups. To ensure that these subjects were in healthy and optimal condition, we excluded pregnant women who had twins (n = 18), delivered preterm (n = 42), or had a history of systematic and chronic diseases such as gestational diabetes mellitus (n = 49), hypertension or preeclampsia (n = 5), and thyroid disease (n = 15). No subject had a stillbirth or a severe postpartum hemorrhage. After exclusion, we were left with 1,055 subjects. We then excluded 62 women whose phlebotomies were not performed in weeks 28 to 40 of the third trimester. After the exclusion process, we were finally left with 993 eligible subjects whose pregnancies were uncomplicated pregnancies and whose laboratory data was complete for weeks 28 to 40 to include in our analysis (Fig 1). All participants provided signed informed consent and procedures were performed in accordance with the principles outlined in the Declaration of Helsinki. This study was approved by the Ethics Committees of National Health Research Institutes (EC1010501) and the respective IRBs of the nine hospitals (S6 Table).

### Laboratory testing

We performed laboratory analyses of all blood samples provided by eligible subjects. Fasting was not required because most of women were unable or unwilling to tolerate fasting due to their pregnancies. All clinical biochemical tests were analyzed in the Union Clinical Laboratory in Taipei, a facility officially accredited by the Taiwan Accreditation Foundation using the same criteria established by ISO 15189: 2012, in effect between 27 June 2018 and 26 June 2021 (Certificate No. L1447–190717) and by the College of American Pathologists‘ Laboratory Accreditation Program in effect prior to 25 January 2022 (CAP number: 6979606). The analytes examined, equipment used, assay coefficients of variation, type of reagent and nomenclature for properties and units (NPU) codes are listed in S1 Table. Because some analytes used Logical Observation Identifiers Names and Codes (LOINC), we also provided them in place of NPU codes [15]. At that time, the Union Clinical Laboratory provided reference intervals of all laboratory analytes for healthy adults, including both men and non-pregnant women but only some of the analytes for pregnant women (Table 2, S2 Table).

A few eligible pregnant women (n = 23) from southern Taiwan provided blood samples after their delivery. We also analyzed their samples using the same sample-taking and laboratory procedures to compare the same subjects in their third trimester and post delivery.

Routine blood tests (hematology) included white blood cell (WBC), red blood cell (RBC), platelet, hemoglobin, hematocrit, mean corpuscular volume (MCV), mean corpuscular hemoglobin (MCH), mean corpuscular hemoglobin concentration (MCHC), neutrophil, lymphocyte, monocyte, eosinophil, and basophil were analyzed by the XN-9000 & Microscopy. Laboratory tests for biochemical indicators including aspartate aminotransferase (AST), alanine aminotransferase (ALT), creatinine (Cr), and random blood sugar were measured by the ADVIA Chemistry XPT. Insulin, thyroid hormones including thyroxine (T4), triiodothyronine (T3), thyroid-stimulating hormone (TSH), and free thyroxine (Free T4), and sex hormones including estradiol, testosterone, progesterone, follicle-stimulating hormone (FSH), and luteinizing hormone (LH) were measured using ADVIA Centaur XPT. Because most FSH (86.61%) and LH values (91.24%) were below detection limits (mIU/mL), 0.30 and 0.07, respectively, these two parameters are not shown.

### Statistical methods

Descriptive statistics were used to summarize characteristics of eligible subjects and their newborns. For biochemical parameters, RI, mean ± standard deviation, and median (IQR: interquartile range) were presented, when appropriate. To calculate the RI percentile (2.5% and 97.5%) of each biochemical parameter for the third trimester, we followed the approach recommended by International Federation of Clinical Chemistry and Laboratory Medicine (IFCC) [16]. For each biochemical parameter, we checked the missing data and outliers according to Horn [17], where the upper and lower outliers are Q3 (the third quartile) + 1.5 IQR (interquartile of range) and Q1 (the first quartile) − 1.5 IQR (S3 Table).

To estimate 90% overall confidence envelopes for the 2.5% and 97.5% conditional quantile functions, a non-parametric bootstrap procedure with 1,000 resamples was used [18].

The envelopes were compared with the corresponding 90% confidence intervals (CIs) of the conventional RI (reference interval) to decide whether and, if so, when the pregnancy-dependent interval became approximately significantly different. An IFCC-recommended approach was used to calculate RI including 90% CI for the limits of the observational period [16]. If a value was biologically unexplainable, suggesting measurement failure, it was removed from the analysis. When a concentration measured below the lower detectable limit of the assay, a value equaling half the detection limit was entered as a test result.

Wilcoxon rank sum test was used to compare the differences of biochemical parameters between group of the first four weeks (weeks 28 to 31) and group of the last four weeks (weeks 37 to 40) of third trimester. Wilcoxon signed-rank test was used to compare biochemical parameters in the pregnant women (n = 23) between the trimester and after delivery. Benjamin-Hochberg method (false discovery rate) was used to avoid the issue of multiple comparisons and decrease the occurrence of type 1 error. False discovery rate was analyzed using the R software version 3.6.3, whereas all other statistical operations were performed using the IBM SPSS version 18.5. A p-value of < 0.05 was considered significant.

## Results

### Demographic characteristics

For 993 eligible subjects included in this study, the mean maternal age at delivery was 31.59 ± 4.46 years, and mean maternal body mass index (kg/m^2^) was 21.76 ± 3.72 before pregnancy and 25.56 ± 3.89 after pregnancy (Table 1). Forty percent (40.42%) of the mothers were primipara and 80.45% had higher education levels (≥ Post-secondary education). Mean gestational age at delivery was 38.72 ± 1.0 weeks. Almost fifty percent (49.04%) of the newborns were male (Table 1).

**Table 1:** Characteristics of eligible pregnant women and their newborns (n = 993).

Variables		N	Mean±SD or %
Mother			
Age, y		893	31.59 ± 4.46
Height, cm		958	159.90 ± 5.30
Weight before pregnancy, kg		943	55.77 ± 10.41
Weight after pregnancy, kg		973	65.56 ± 10.95
BMI (before pregnancy), kg/m^2^		935	21.76 ± 3.72
BMI (after pregnancy), kg/m^2^		953	25.56 ± 3.89
Primipara		363	40.42%
Education level	< Post-secondary education	193	19.55%
	≥ Post-secondary education	794	80.45%
Newborn			
Gender*	Male	487	49.04%
	Female	424	42.70%
Gestational age, week		902	38.72 ± 1.00
Birth length, cm		884	49.53 ± 2.38
Birth weight, g		901	3128.50 ± 370.94
Head circumference, cm		845	33.70 ± 1.35
Chest circumference, cm		838	32.42 ± 1.54

*Lack of gender information in 82 newborns.

### Reference intervals

Table 2 summarizes the third trimester laboratory values that clinical investigators and most clinicians might need when caring for pregnant women after we excluded missing data and outliers (listed in S3 Table). Compared to the RIs for general adults provided by the Union Clinical Laboratory, pregnant women in their third trimester had increases in both lower and upper normal limits for neutrophil, T3, T4, testosterone, estradiol, and progesterone, and had decreases in the normal lower and upper limits for RBC, Hb, HCT, MCH, MCHC, platelet, lymphocyte, ALT and creatinine (Table 2, S2 Table).

**Table 2:** The comparison between Reference Intervals (RI) of third trimester of pregnant women in Taiwan (TMICS) and those of general adults obtained from The Union Clinical Laboratory (ISO 15189:2007).

Item	Unit	n	Median	3rd trimester		Union Clinical Laboratory (General adults)*	
				RI percentile		RI percentile	
				2.5^th^	97.5^th^	2.5^th^	97.5^th^
**Hematology**							
White blood cell	10^3^/uL	913	8.20	3.10	12.92	3.5	10
Red blood cell	10^6^/uL	913	3.90	3.27	4.67	3.7	5.5
Hemoglobin	g/dL	924	11.50	9.10	13.70	11.3	15.3
Platelet	10^3^/uL	916	213.50	106.85	332.00	150	400
**Biochemical indicators**							
Aspartate aminotransferase	U/L	906	21.00	13.00	34.00	10	42
Alanine aminotransferase	U/L	921	15.00	7.00	25.00	10	40
Creatinine	mg/dL	931	0.51	0.38	0.75	0.5	1.1
Insulin	mIU/L	898	16.00	2.00	84.91	3	25
Random blood sugar	mg/dL	809	86.00	63.25	126.00		AC:70–100
							PC:70–139
**Thyroid hormones**							
Triiodothyronine	ng/dL	963	129.00	82.10	190.90	60	181
Thyroxine	ug/dL	967	11.10	7.70	14.30	4.5	10.9
Free thyroxine	ng/dL	969	0.98	0.77	1.24	0.74	1.47
Thyroid-stimulating hormone	uIU/mL	928	1.38	0.40	3.33	0.55	4.78
**Sex hormones**							
Testosterone	ng/dL	942	81.00	36.00	176.43	14	76
Estradiol	pg/mL	906	14571.80	5754.97	28036.96		Follicular phase: 19.5–144.2
							Ovulation phase: 63.9–356.7
							Luteal phase: 55.8–214.2
Progesterone	ng/mL	937	129.10	53.58	292.70		Follicular: ND-1.40
							Luteal: 3.34–25.56
							Mid Luteal: 4.44–28.03
							Postmeopausal: ND-0.73
							Pregnancy
							1st trimester: 11.22–90.0
							2nd trimester: 25.55–89.40
							3rd trimester: 48.40–422.50
Follicle stimulating hormone (FSH)	mIU/mL	86.61% < Detection limit (0.3 mIU/mL)					Follicular phase: 2.5–10.2
							Ovulation phase: 3.4–33.4
							Luteal phase: 1.5–9.1
							Pregnancy: 0.0–0.3
Luteinizing hormone (LH)	mIU/mL	91.24% < Detection limit (0.07 mIU/mL)					Follicular phase: 1.9–12.5
							Ovulation phase: 8.7–76.3
							Luteal phase: 0.5–16.9
							Pregnancy: < 0.1–1.5

*General population includes men and non-pregnant women. Union Clinical Laboratory also provided RI for progesterone, FSH, and LH for women during pregnancy, data from https://www.ucl.com.tw/webshop/shop/ accessed on 09/15/2020.

**Fig 2:**
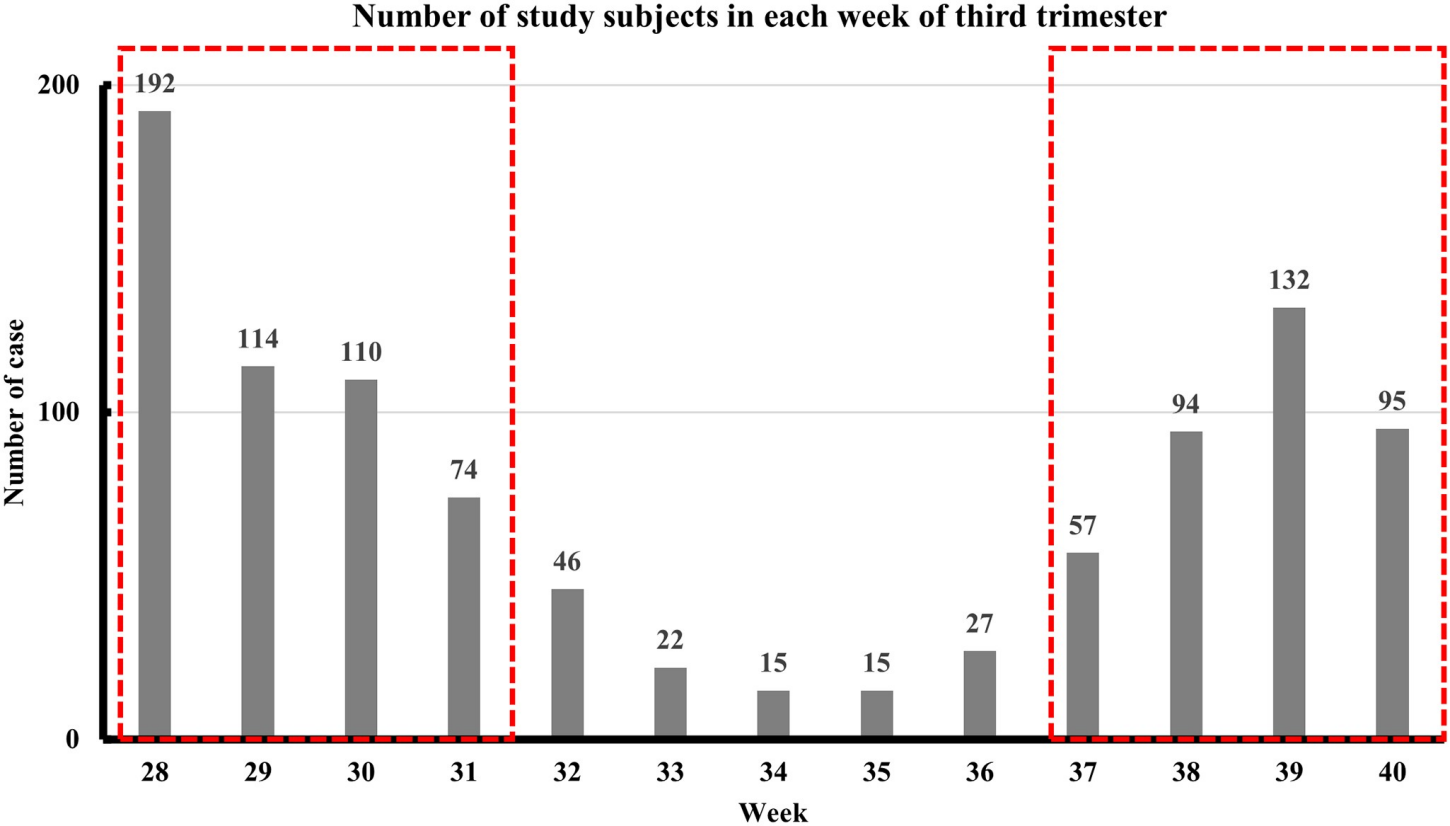
Number of phlebotomies and laboratory tests in the 3rd trimester pregnancy (n = 993).

Compared to the median values for women in their early third trimester (weeks 28 to 31) (n = 490), women in their late third trimester (weeks 37 to 40) (n = 378) had significantly higher median RBC, Hb, Hct, AST, Cr, TSH, testosterone, estradiol, and progesterone and significantly lower median platelets and insulin (Fig 2; Table 3; S5 Table). There were no significant early-late trimester changes in WBC, ALT, sugar, T3, T4, and free T4 (Table 3; S5 Table). We also compared third trimester and post-delivery data obtained from 23 women. The women had lower median AST, ALT, insulin, T3, T4, testosterone, estradiol, and progesterone after delivery and higher median Cr, free T4, FSH, and LH (Table 4). There was almost no change in serum TSH between the two periods.

**Table 3:** Comparison of reference intervals between the early third trimester (weeks 28 to 30) and the late third trimester (weeks 37 to 40) in pregnant women.

Item	Unit	Between 28 and 31				Between 37 and 40				*P value	False discovery rate
		n	Median	2.5th	97.5th	N	Median	2.5th	97.5th		
**Hematology**											
White blood cell	10^3^/uL	467	8.20	2.87	12.93	327	8.20	3.54	13.38	0.89	0.93
Red blood cell	10^6^/uL	469	3.80	3.23	4.68	325	4.06	3.31	4.67	< 0.001	< 0.002
Hemoglobin	g/dL	470	11.40	9.30	13.32	336	11.70	8.78	13.96	< 0.001	< 0.002
Platelet	10^3^/uL	466	223.00	105.05	335.98	333	205.00	100.35	329.25	< 0.001	< 0.002
**Biochemical indicators**											
Aspartate aminotransferase	U/L	473	20.00	13.00	30.15	311	23.00	14.00	36.00	< 0.001	< 0.002
Alanine aminotransferase	U/L	461	14.00	7.00	25.00	340	15.00	7.00	26.00	0.23	0.30
Creatinine	mg/dL	483	0.49	0.38	0.69	326	0.56	0.39	0.77	< 0.001	< 0.002
Insulin	mIU/L	434	18.80	3.25	87.36	348	12.20	0.97	82.58	< 0.001	< 0.002
Random blood sugar	mg/dL	415	86.00	63.00	127.00	281	87.00	59.20	125.00	0.58	0.63
**Thyroid hormones**											
Triiodothyronine	ng/dL	479	130.00	85.00	195.00	363	127.00	77.10	181.00	0.11	0.15
Thyroxine	ug/dL	479	11.10	7.50	14.40	366	11.00	7.70	14.25	0.52	0.59
Free thyroxine	ng/dL	480	0.97	0.77	1.25	366	0.99	0.75	1.24	0.35	0.44
Thyroid stimulating hormone	uIU/mL	480	1.22	0.32	2.98	326	1.80	0.38	3.62	< 0.001	< 0.002
**Sex hormones**											
Testosterone	ng/dL	484	69.00	33.13	143.75	336	114.00	52.00	188.00	< 0.001	< 0.002
Estradiol	pg/mL	472	13226.25	6106.52	23691.63	312	18684.15	3155.56	29125.76	< 0.001	< 0.002
Progesterone	ng/mL	476	101.62	55.52	206.30	339	186.42	28.97	313.07	< 0.001	< 0.002

**Table 4:** Comparison of reference intervals between the third trimester of pregnant women and after delivery (n = 23).

	3rd trimester (a)		After delivery (b)		Difference (b-a)			
	median	25th ~ 75th	median	25th ~ 75th	median	25th ~ 75th	* P value	FDR
Aspartate aminotransferase	20.00	17.00 ~ 24.00	15.00	12.00 ~ 15.00	-7.00	-9.00 ~ -2.00	0.001	0.001
Alanine aminotransferase	15.00	11.00 ~ 18.00	8.00	7.00 ~ 11.00	-7.00	-9.00 ~ -2.00	0.001	0.001
Creatinine	0.54	0.47 ~ 0.57	0.79	0.73 ~ 0.83	0.26	0.21 ~ 0.32	< 0.001	< 0.001
Insulin	25.60	11.20 ~ 106.70	6.90	5.00 ~ 12.10	-16.90	-98.30 ~ -4.20	< 0.001	< 0.001
Triiodothyronine	132.00	113.50 ~ 145.50	100.00	89.50 ~ 116.00	-30.00	-42.00 ~ -5.00	0.003	0.003
Thyroxine	11.20	9.70 ~ 12.80	7.60	6.80 ~ 8.50	-3.50	-4.30 ~ -2.50	< 0.001	< 0.001
Free thyroxine	1.03	0.89 ~ 1.08	1.13	1.00 ~ 1.21	0.13	0.06 ~ 0.26	0.001	0.001
Thyroid stimulating hormone	1.66	1.26 ~ 2.23	1.29	0.99 ~ 2.44	-0.24	-1.07 ~ 0.33	0.25	0.25
Testosterone	92.00	70.00 ~ 122.00	23.54	16.42 ~ 26.41	-75.50	-95.66 ~ -43.59	< 0.001	< 0.001
Estradiol	14543.95	11982.18 ~ 17783.93	108.85	51.25 ~ 239.25	-14463.90	-17725.40~-11922.68	< 0.001	< 0.001
Progesterone	146.36	102.55 ~ 202.53	0.93	0.24 ~ 11.00	-129.30	-189.22 ~ -91.55	< 0.001	< 0.001
Follicle stimulating hormone	0.15	0.15 ~ 0.15	3.95	2.48 ~ 7.48	3.80	2.33 ~ 7.33	< 0.001	< 0.001
Luteinizing hormone	0.04	0.04 ~ 0.04	3.93	2.16 ~ 5.00	3.90	2.13 ~ 4.97	< 0.001	< 0.001

* P values were tested using Wilcoxon sign rank test.

§Values below the lowest detection limit were defined as half of lowest detection limit, whereas values above the highest detection limit were defined as the highest detection limit. Thus, the values were 0.15 for FSH < 0.3, 0.035 for LH < 0.07, 0.105 for progesterone < 0.21, 5.9 for estradiol < 11.8, and 3.5 for testosterone < 7, whereas the value was 3,000 for estradiol > 3,000.

## Discussion

This study is the first nationwide cohort study to establish RIs of important biochemical parameters for pregnant Taiwanese women in their third trimester. Many common hormone level change dramatically during pregnancy. Sex hormones, including estradiol, progesterone, and testosterone, all rise markedly at this time, as do lower and upper limits for neutrophil, T3, and T4 during the third trimester. Hematological values remain relatively stable. Plasma volume increases by 10–15% at 6–12 weeks of gestation [19], and red cell mass increases, though not as much, leading to dilutional anemia and a 1–2 g/dL drop in hemoglobin [20]. Platelets decrease during pregnancy, a phenomenon known as “gestational thrombocytopenia”, possibly a result of hemodilution, increased platelet activation, and accelerated clearance [21]. Leucocytosis, an increase in leucocytes, also occurs during pregnancy [22], and so do Neutrophils, which are major leucocytes, because neutrophilic apoptosis is impaired during this time [23]. In addition, lymphocytes decrease during the first and second trimesters of pregnancy and then increase during the third trimester [20].

Overall, the current study found hematology ranges that were similar to previous studies. Neutrophils were slightly higher in our cohort than in the general adult population. During their third trimester, our cohort had slightly lower HB, HCT, MCH, MCHC, platelets, and lymphocytes. Compared to values reported for the same trimester in a reference paper, our third trimester RBC and HCT ranges were slightly higher and our WBC, HB, and platelet ranges were slightly lower [8].

With regard to the sex hormones, pregnant women in their third trimester had much higher median testosterone and estradiol RIs than the general population (Table 2). Almost all (91.24%) of their LH values were below lowest detection limit for LH (0.07 mIU/Ml), a finding similar to that of the Union Clinical Laboratory in Taiwan (< 0.1–1.5 mIU/mL during pregnancy) (Table 2). Estradiol, which increases due to placental production, stimulates uterine blood flow, myometrial growth, and breast growth [24]. Abbassi-Ghanavati et al., referencing studies found in PubMed and Medline 1975–2008 and textbooks, assembled a table of normal trimester ranges [8]. The testosterone levels and the upper limit of the progesterone rage were 0.57 and 0.86 times lower, respectively than they reported. The upper limit of our cohort’s estradiol range was much higher than theirs but lower than that reported in O’Leary et al. [25], possibly due to racial or regional differences or sampling times. Another possible reason for the difference was that some of the values Abbassi-Ghanavati et al. published were obtained from two or more studies whose samples were analyzed by different laboratories that may evaluated the analytes using different assays.

We also compared our RIs for other biochemical parameters with those reported by other studies. Several relevant studies have recently provided RIs for clinical biochemical parameters in pregnant women in China [4, 5, 12]. Two of those studies found pregnant women in their third trimester had lower Cr compared to non-pregnant women [4, 12], similar to our findings. There has been no agreement on the effect of pregnancy on serum AST and ALT. Some studies, like ours, report a slight increase in AST and/or ALT levels in the third trimester [26, 27], while other studies found slight decreases [4, 12], the differences possibly due to racial make up of the populations they studied, underlying liver diseases, or other factors. Some studies of a Chinese population report RBC, Hb, HCT, MCHC, lymphocyte count, and PLT to be lower and WBC and neutrophil counts to be higher in pregnant women [4, 5], as we have. With regard to thyroid function, Moon et al. reported significant increases in median TSH levels and significant decreases in median free T4 levels over the different trimesters in pregnant women in Korea [3]. Their upper and lower limits for TSH and free T4 levels in the third trimester were lower than those of non-pregnant women [3]. Similarly, we found upper and lower normal limits of TSH to be lower and the upper limit for free T4 to be slightly lower during the third trimester of pregnant women. These findings indicate that studies conducted in different countries generate different RIs references over the trimesters, underscoring the importance of establishing RIs for our own population.

Pregnant women in their late third trimester had higher median RBC, Hb, AST, Cr, TSH, testosterone, estradiol, and progesterone and lower median platelet counts and insulin than those in their early third trimester, suggesting a need to establish RIs for these two periods separately. We found a slight increase in RBC and Hb in the late trimester. This finding was similar to that of a previous study that suggested this difference may be due to the common use of oral iron supplement by pregnant women during their late trimester [10, 28]. We found a lower platelet count in the late third trimester, which according one previous study, results from gestational thrombocytopenia [21]. The increase in AST levels in the late third trimester might be caused by the contraction of the uterine muscles [26]. We found a mild elevation in serum creatinine in late third trimester, similar to a previous study [29]. According another study, this mild elevation is related to effective renal blood flow which varies during pregnancy [30]. Our findings of higher TSH, testosterone, estradiol, and progesterone levels in the late third trimester are compatible with previous studies [3, 10, 25]. We found insulin levels to be lower in the late third trimester, compared with the early third trimester, possibly caused in part by hormones from the placenta and in part by body weight and other pregnancy-related factors [31].

Few studies have compared RIs during and after pregnancy. We compared third trimester values with post-delivery values and found women after delivery had lower normal lower and upper limits for AST, ALT, Insulin, T3, T4, testosterone, estradiol, and progesterone and higher normal lower and upper limits for Cr, free thyroxine, FSH, and LH than women in their third trimester (Table 4). Post-delivery sex hormones, including testosterone, estradiol, and progesterone, were all lower due to removal of the placenta, and FSH and LH levels were higher, increased by negative feedback control mechanisms related to the hypothalamic-pituitary-gonadal axis [25, 32]. GFR levels are normally elevated by 40% throughout pregnancy and drop to levels found in normal non-pregnant women within one month after delivery [33]. Because serum Cr level is in inverse proportion to GFR based on the equation of Modification of Diet in Renal Disease (MDRD) [34], we also found that the upper and lower limits for Cr increased after delivery, compared to the third trimester. Our findings were similar. Once a baby is delivered, the glycaemic and insulin levels increased by pregnancy, are reduced [35, 36], as they were in our study. In addition, post-delivery median T3 and T4 were lower and free T4 slightly were higher due to decreases hCG and TBG after delivery [37, 38].

This study is unique in that it is the first nationwide birth cohort study whose main goal was to establish reference intervals of important biochemical parameters in the third trimester and the first in Taiwan to establish those for early and late third trimester periods. Because our sample sources resided in northern, central, southern and eastern Taiwan, our study’s reference values can be considered representative. This study is one of the very few studies with large enough sample sizes to attempt to establish reference intervals for sex hormones in women during their trimester of pregnancy. Our blood samples were all tested in the same central clinical laboratory, so interference from differences in operators and reagents were avoided.

This study has some limitations. One limitation is that it only focused on the establishment of the normal ranges during the third trimester, so we lacked data for first and second trimesters. Another limitation is that our subjects were not randomly selected, so there may have been some selection bias. The hospitals in our study were all medical centers, which might attract more health-conscious patients or women with higher economic status or may simply attract people living nearby.

## Conclusions

In conclusion, we believe that the table of reference intervals of important parameters presented in this study can serve as a quick reference for clinicians caring for pregnant women and newborn infants. The reference values for many common hormones, especially estradiol, progesterone, and testosterone, shift during pregnancy. Gestation-specified values should be considered by any clinician when evaluating laboratory values in a pregnant woman because there may be some risk of misinterpreting physiological adaptations of pregnancy as being pathologic or some risk that they mask other pathologies.

## Supporting information

S1 Table Details of blood analytes from the pregnant women during their third trimester. (DOC)

S2 Table Reference Intervals (RI) of other biochemical parameters in the third trimester of pregnant women in Taiwan (TMICS) and general adults from the Union Clinical Laboratory (ISO 15189:2007). (DOC)

S3 Table Number, missing data, and outliers of the third trimester of pregnant women in Taiwan (TMICS). (DOC)

S4 Table 90% CI for the Reference Intervals (RI) of pregnant women during their third trimester in Taiwan (TMICS). (DOC)

S5 Table Comparison of reference intervals between the first four weeks (weeks 28 to 31) and the last four weeks (weeks 37 to 40) of the third trimester of pregnant women. (DOC)

S6 Table The approval IRB numbers from National Health Research Institutes (NHRI) and other nine hospitals. (DOC)

S1 Dataset (XLSX)

## Abbreviations

ALT: alanine aminotransferase
AST: aspartate aminotransferase
CIs: confidence intervals
Cr: creatinine
DEHP: di-(2-ethylhexyl) phthalate
DHEA-S: dehydroepiandrosterone sulfate
E2: estradiol
free T4: free thyroxine
FSH: follicle-stimulating hormone
GFR: Glomerular filtration rate
HB: hemoglobin
hCG: Human chorionic gonadotropin
HCT: hematocrit
IFCC: International Federation of Clinical Chemistry and Laboratory Medicine
INF-γ: interferon gamma
IQR: interquartile of range
LH: luteinizing hormone
LOINC: Logical Observation Identifiers Names and Codes
MCH: mean corpuscular hemoglobin
MCHC: mean corpuscular hemoglobin concentration
MCV: mean corpuscular volume
MDRD: Modification of Diet in Renal Disease
NPU: Nomenclature for Properties and Units
PAPP-A: pregnancy associated plasma protein-A
Q1: the first quartile
Q3: the third quartile
RBC: red blood cell
RI: reference interval
SD: standard deviation
T3: triiodothyronine
T4: thyroxine
TBG: thyroid binding globulin
TESBIO: bioavailable testosterone
TMICS: Taiwan Maternal and Infant Cohort Study
TNF-α: tumor necrosis factor-α
TSH: thyroid-stimulating hormone
TT: testosterone
WBC: white blood cell

## References

[1] Xing J, Yuan E, Li J, Zhang Y, Meng X, Zhang X, et al Trimester- and Assay-Specific Thyroid Reference Intervals for Pregnant Women in China. Int J Endocrinol. 2016;2016:3754213 10.1155/2016/3754213 . 27087808 PMC4819108

[2] Nazarpour S, Ramezani Tehrani F, Simbar M, Minooee S, Rahmati M, Mansournia MA, et al Establishment of trimester-specific reference range for thyroid hormones during pregnancy. Clin Biochem. 2018;53:49–54. 10.1016/j.clinbiochem.2018.01.006 . 29337034

[3] Moon HW, Chung HJ, Park CM, Hur M, Yun YM. Establishment of trimester-specific reference intervals for thyroid hormones in Korean pregnant women. Ann Lab Med. 2015;35(2):198–204. 10.3343/alm.2015.35.2.198 . 25729721 PMC4330169

[4] Jin Y, Lu J, Jin H, Fei C, Xie X, Zhang J. Reference intervals for biochemical, haemostatic and haematological parameters in healthy Chinese women during early and late pregnancy. Clin Chem Lab Med. 2018;56(6):973–9. 10.1515/cclm-2017-0804 . 29303769

[5] Li A, Yang S, Zhang J, Qiao R. Establishment of reference intervals for complete blood count parameters during normal pregnancy in Beijing. J Clin Lab Anal. 2017;31(6). 10.1002/jcla.22150 . 28105762 PMC6816986

[6] Sharma K, Singh R, Kumar M, Gupta U, Rohil V, Bhattacharjee J. First-Trimester Inflammatory Markers for Risk Evaluation of Pregnancy Hypertension. J Obstet Gynaecol India. 2018;68(1):27–32. 10.1007/s13224-017-0988-1 29391672 PMC5783907

[7] Wickstrom K, Edelstam G, Lowbeer CH, Hansson LO, Siegbahn A. Reference intervals for plasma levels of fibronectin, von Willebrand factor, free protein S and antithrombin during third-trimester pregnancy. Scand J Clin Lab Invest. 2004;64(1):31–40. 10.1080/00365510410003859 . 15029874

[8] Abbassi-Ghanavati M, Greer LG, Cunningham FG. Pregnancy and laboratory studies: a reference table for clinicians. Obstet Gynecol. 2009;114(6):1326–31. 10.1097/AOG.0b013e3181c2bde8 . 19935037

[9] Larsson A, Palm M, Hansson LO, Axelsson O. Reference values for clinical chemistry tests during normal pregnancy. BJOG. 2008;115(7):874–81. 10.1111/j.1471-0528.2008.01709.x . 18485166

[10] Klajnbard A, Szecsi PB, Colov NP, Andersen MR, Jorgensen M, Bjorngaard B, et al Laboratory reference intervals during pregnancy, delivery and the early postpartum period. Clin Chem Lab Med. 2010;48(2):237–48. 10.1515/CCLM.2010.033 . 19943809

[11] Friis Petersen J, Friis-Hansen LJ, Jensen AK, Nyboe Andersen A, Lokkegaard ECL. Early pregnancy reference intervals; 29 serum analytes from 4 to 12 weeks’ gestation in naturally conceived and uncomplicated pregnancies resulting in live births. Clin Chem Lab Med. 2019;57(12):1956–67. 10.1515/cclm-2019-0495 . 31343977

[12] Dai Y, Liu J, Yuan E, Li Y, Wang Q, Jia L, et al Gestational age-specific reference intervals for 15 biochemical measurands during normal pregnancy in China. Ann Clin Biochem. 2018;55(4):446–52. 10.1177/0004563217738801 29153025

[13] Wu CF, Chen HM, Sun CW, Chen ML, Hsieh CJ, Wang SL, et al Cohort Profile: The Taiwan Maternal and Infant Cohort Study (TMICS) of phthalate exposure and health risk assessment. Int J Epidemiol. 2018;47(4):1047–j. 10.1093/ije/dyy067 . 29718277

[14] Wu MT, Wu CF, Wu JR, Chen BH, Chen EK, Chao MC, et al The public health threat of phthalate-tainted foodstuffs in Taiwan: the policies the government implemented and the lessons we learned. Environ Int. 2012; 44: 75–79. 10.1016/j.envint.2012.01.014 . 22361240

[15] McDonald CJ, Huff SM, Suico JG, Hill G, Leavelle D, Aller R, et al LOINC, a universal standard for identifying laboratory observations: a 5-year update. Clin Chem. 2003;49(4):624–33. 10.1373/49.4.624 . 12651816

[16] Solberg HE. The IFCC recommendation on estimation of reference intervals. The RefVal program. Clin Chem Lab Med. 2004;42(7):710–4. 10.1515/CCLM.2004.121 . 15327004

[17] Horn PS, Feng L, Li Y, Pesce AJ. Effect of outliers and nonhealthy individuals on reference interval estimation. Clin Chem. 2001;47(12):2137–45. . 11719478

[18] Davison AC, Hinkley DV. Bootstrap methods and their application. Cambridge; New York, NY, USA: Cambridge University Press; 1997 x, 582 p. p.

[19] Bernstein IM, Ziegler W, Badger GJ. Plasma volume expansion in early pregnancy. Obstet Gynecol. 2001;97(5): 669–672. 10.1016/s0029-7844(00)01222-9 . 11339913

[20] Chandra S, Tripathi AK, Mishra S, Amzarul M, Vaish AK. Physiological changes in hematological parameters during pregnancy. Indian J Hematol Blood Transfus. 2012;28(3):144–6. 10.1007/s12288-012-0175-6 . 23997449 PMC3422383

[21] Shehata N, Burrows R, Kelton JG. Gestational thrombocytopenia. Clin Obstet Gynecol. 1999;42(2):327–34. 10.1097/00003081-199906000-00017 . 10370851

[22] Pitkin RM, Witte DL. Platelet and leukocyte counts in pregnancy. JAMA. 1979;242(24):2696–8. . 501868

[23] Gatti L, Tenconi PM, Guarneri D, Bertulessi C, Ossola MW, Bosco P, et al Hemostatic parameters and platelet activation by flow-cytometry in normal pregnancy: a longitudinal study. Int J Clin Lab Res. 1994;24(4):217–9. 10.1007/BF02592466 . 7894047

[24] Picciano MF. Pregnancy and lactation: physiological adjustments, nutritional requirements and the role of dietary supplements. J Nutr. 2003;133(6):1997S–2002S. 10.1093/jn/133.6.1997S . 12771353

[25] O’Leary P, Boyne P, Flett P, Beilby J, James I. Longitudinal assessment of changes in reproductive hormones during normal pregnancy. Clin Chem. 1991;37(5):667–672. 1827758

[26] Gohel MG, Joshi AG, Anand JS, Makadia JS, Kamariya CP. Evaluation of changes in liver function test in first, second and third trimester of normal pregnancy. Int J Reprod Contracep Obstet Gynecol. 2013;2(4):616–620.

[27] Mutua DN, Njagi ENM, Orinda G. Liver Function Tests in Normal Pregnant Women. J Liver. 2018;7(2):1000228.

[28] Milman N, Bergholt T, Byg KE, Eriksen L, Graudal N. Iron status and iron balance during pregnancy. A critical reappraisal of iron supplementation. ACTA OBSTET GYN SCAN 1999;78(9): 749–757. 10535335

[29] Harel Z, McArthur E, Hladunewich M, Dirk JS, Wald R, Garg AX, et al Serum creatinine levels before, during, and after pregnancy. JAMA. 2019;321(2): 205–207. 10.1001/jama.2018.17948 30644975 PMC6439761

[30] Davison J, Dunlop W. Changes in renal hemodynamics and tubular function induced by normal human pregnancy. SEMIN NEPHROL. 1984;4(3):198–207.

[31] Kampmann U, Knorr S, Fuglsang J, Ovesen P. Determinants of Maternal Insulin Resistance during Pregnancy: An Updated Overview. J Diabetes Res. 2019; 11 19;2019:5320156 10.1155/2019/5320156 . 31828161 PMC6885766

[32] Stricker R, Eberhart R, Chevailler MC, Quinn FA, Bischof P, Stricker R. Establishment of detailed reference values for luteinizing hormone, follicle stimulating hormone, estradiol, and progesterone during different phases of the menstrual cycle on the Abbott ARCHITECT analyzer. Clin Chem Lab Med. 2006; 44(7): 883–87. 10.1515/CCLM.2006.160 . 16776638

[33] Krutzen E, Olofsson P, Bäck SE, Nilsson-Ehle P. Glomerular filtration rate in pregnancy: a study in normal subjects and in patients with hypertension, preeclampsia and diabetes. Scand J Clin Lab Invest. 1992;52(5): 387–92. 10.3109/00365519209088374 . 1514017

[34] Levey AS, Coresh J, Greene T, Stevens LA, Zhang YL, Hendriksen S, et al Using standardized serum creatinine values in the modification of diet in renal disease study equation for estimating glomerular filtration rate. Ann Intern Med. 2006;145(4): 247–54. 10.7326/0003-4819-145-4-200608150-00004 . 16908915

[35] Ciampelli M, Lanzone A, Caruso A. Insulin in obstetrics: a main parameter in the management of pregnancy. Hum Reprod Update. 1998;4(6):904–14. 10.1093/humupd/4.6.904 . 10098480

[36] Spellacy WN, Goetz FC. Plasma insulin in normal late pregnancy. N Engl J Med. 1963;268:988–91. 10.1056/NEJM196305022681805 . 13989996

[37] Costantine MM. Physiologic and pharmacokinetic changes in pregnancy. Front Pharmacol. 2014;5:65 10.3389/fphar.2014.00065 . 24772083 PMC3982119

[38] Glinoer D. The regulation of thyroid function in pregnancy: pathways of endocrine adaptation from physiology to pathology. Endocr Rev. 1997;18(3):404–33. 10.1210/edrv.18.3.0300 . 9183570

