# Supplementary material for: New trimester-specific reference intervals for clinical biochemical tests in Taiwanese pregnant women-cohort of TMICS
Source: PLoS One. 2020 Dec 14;15(12):e0243761. doi: 10.1371/journal.pone.0243761 (PMC7735596; doi:10.1371/journal.pone.0243761)
Supplement: S1 Table — (DOC) [file pone.0243761.s001.doc]

**S1 Table. Details of blood analytes from the pregnant women during their third trimester.**

| **Analyte (abbreviation)** |  | **Equipment** | **CV%** | **Type of reagent** | **NPU code** | **LOINC code** |
| --- | --- | --- | --- | --- | --- | --- |
| **Hematology** |  |  | | | | |
| White blood cell |  | XN-9000 & Microscopy | - | EDTA | NPU02593 | 06690-2 |
| Red blood cell |  | XN-9000 & Microscopy | - | EDTA | NPU17564 | 789-8 |
| Hemoglobin |  | XN-9000 & Microscopy | - | EDTA | NPU28309 | 718-7 |
| Hematocrit |  | XN-9000 & Microscopy | - | EDTA | NPU01961 | 20570-8 |
| Mean corpuscular volume |  | XN-9000 & Microscopy | - | EDTA | NPU01944 | 30428-7 |
| Mean corpuscular hemoglobin |  | XN-9000 & Microscopy | - | EDTA | NPU26880 | 785-6 |
| Mean corpuscular hemoglobin concentration |  | XN-9000 & Microscopy | - | EDTA | NPU28315 | 786-4 |
| Platelet |  | XN-9000 & Microscopy | - | EDTA | None | 777-3 |
| Neutrophil |  | XN-9000 & Microscopy | - | EDTA | None | 770-8 |
| Lymphocyte |  | XN-9000 & Microscopy | - | EDTA | None | 26478-8 |
| Monocyte |  | XN-9000 & Microscopy | - | EDTA | None | 744-3 |
| Eosinophil |  | XN-9000 & Microscopy | - | EDTA | None | 26450-7 |
| Basophil |  | XN-9000 & Microscopy | - | EDTA | None | 71675-3 |
| **Biochemical indicators** |  |  | | | | |
| Aspartate aminotransferase |  | ADVIA Chemistry XPT | - | - | NPU57159 | 01920-8 |
| Alanine aminotransferase |  | ADVIA Chemistry XPT | - | - | NPU53495 | 77144-4 |
| Creatinine |  | ADVIA Chemistry XPT | - | - | NPU04998 | 11041-1 |
| Insulin |  | ADVIA CentaurXPT | - | - | NPU56319 | 20448-7 |
| Random blood sugar |  | ADVIA Chemistry XPT | - | NaF | NPU10119 | 74774-1 |
| **Thyroid hormones** |  |  | | | | |
| Triiodothyronine |  | ADVIA CentaurXPT | 5.5 | - | NPU03624 | 03053-6 |
| Thyroxine |  | ADVIA CentaurXPT | 4.59 | - | NPU03578 | 03026-2 |
| Free thyroxine |  | ADVIA CentaurXPT | 7.14 | - | NPU03579 | 03024-7 |
| Thyroid-stimulating hormone |  | ADVIA CentaurXPT | - | Heparin or EDTA | NPU56169 | 03016-3 |
| **Sex hormones** |  |  | | | | |
| Testosterone |  | ADVIA Centaur XPT | - | Lithium heparin，Sodium heparin，K2-EDTA | NPU03543 | 02986-8 |
| Estradiol |  | ADVIA CentaurXPT | 7.73 | EDTA and Heparin | NPU01972 | 02243-4 |
| Progesterone |  | ADVIA CentaurXPT | - | - | NPU03242 | 02839-9 |
| Follicle-stimulating hormone |  | ADVIA CentaurXPT | 5.98 | - | None | 15067-2 |
| Luteinizing hormone |  | ADVIA CentaurXPT | 5.26 | - | None | 10501-5 |

**“-“ means no available data**
