# Supplementary material for: New trimester-specific reference intervals for clinical biochemical tests in Taiwanese pregnant women-cohort of TMICS
Source: PLoS One. 2020 Dec 14;15(12):e0243761. doi: 10.1371/journal.pone.0243761 (PMC7735596; doi:10.1371/journal.pone.0243761)
Supplement: S2 Table — (DOC) [file pone.0243761.s002.doc]

| **S2 Table. Reference intervals (RI) of other biochemical parameters in the third trimester of pregnant women in Taiwan (TMICS) and general adults from the Union Clinical Laboratory (ISO 15189:2007).** | | | | | | | | |
| --- | --- | --- | --- | --- | --- | --- | --- | --- |
| **Item** | **Unit** | **n** | **Median** | **3rd trimester** | |  | **Union Clinical Laboratory (General adults)*** | |
|  |  |  |  |  |  |  |  |  |
|  |  |  |  | **RI percentile** | |  | **RI percentile** | |
|  |  |  |  |  |  |  |  |  |
|  |  |  |  | **2.5^th^** | **97.5^th^** |  | **2.5^th^** | **97.5^th^** |
| **Hematology** |  |  |  |  |  |  |  |  |
| Hematocrit | % | 913 | 35.80 | 29.70 | 44.20 |  | 33 | 47 |
| Mean corpuscular volume | fL | 863 | 92.70 | 79.66 | 106.58 |  | 80 | 99 |
| Mean corpuscular hemoglobin | pg | 886 | 30.00 | 24.20 | 33.10 |  | 26 | 34 |
| Mean corpuscular hemoglobin concentration | g/dL | 919 | 32.30 | 27.00 | 34.40 |  | 30 | 36 |
| Neutrophil | % | 861 | 74.90 | 62.00 | 85.10 |  | 39 | 74 |
| Lymphocyte | % | 867 | 18.30 | 10.00 | 29.43 |  | 19 | 48 |
| Monocyte | % | 875 | 4.90 | 2.10 | 8.01 |  | 2 | 10 |
| Eosinophil | % | 860 | 0.95 | 0.20 | 3.00 |  | 0 | 7 |
| Basophil | % | 883 | 0.20 | 0.00 | 0.50 |  | 0 | 1.5 |

*General population indicates men and non-pregnant women.
