# Supplementary material for: New trimester-specific reference intervals for clinical biochemical tests in Taiwanese pregnant women-cohort of TMICS
Source: PLoS One. 2020 Dec 14;15(12):e0243761. doi: 10.1371/journal.pone.0243761 (PMC7735596; doi:10.1371/journal.pone.0243761)
Supplement: S3 Table — (DOC) [file pone.0243761.s003.doc]

**S3 Table. Number, missing data, and outliers of the third trimester of pregnant women in** Taiwan (TMICS).

| **Item** | **Unit** | **N** | **Missing** | **Outlier** | **Total** |  |
| --- | --- | --- | --- | --- | --- | --- |
|  |
|  |
| **Hematology** |  |  |  |  |  |  |
| White blood cell | 10^3^/uL | 913 | 55 | 25 | 993 |  |
| Red blood cell | 10^6^/uL | 913 | 55 | 25 | 993 |  |
| Hemoglobin | g/dL | 924 | 55 | 14 | 993 |  |
| Hematocrit | % | 913 | 55 | 25 | 993 |  |
| Mean corpuscular volume | fL | 863 | 55 | 75 | 993 |  |
| Mean corpuscular hemoglobin | pg | 886 | 55 | 52 | 993 |  |
| Mean corpuscular hemoglobin concentration | g/dL | 919 | 55 | 19 | 993 |  |
| Platelet | 10^3^/uL | 916 | 55 | 22 | 993 |  |
| Neutrophil | % | 861 | 94 | 38 | 993 |  |
| Lymphocyte | % | 867 | 94 | 32 | 993 |  |
| Monocyte | % | 875 | 94 | 24 | 993 |  |
| Eosinophil | % | 860 | 94 | 39 | 993 |  |
| Basophil | % | 883 | 93 | 17 | 993 |  |
| **Biochemical indicators** |  |  |  |  |  |  |
| Aspartate aminotransferase | U/L | 906 | 10 | 77 | 993 |  |
| Alanine aminotransferase | U/L | 921 | 11 | 61 | 993 |  |
| Creatinine | mg/dL | 931 | 6 | 56 | 993 |  |
| Insulin | mIU/L | 898 | 19 | 76 | 993 |  |
| Random blood sugar | mg/dL | 809 | 139 | 45 | 993 |  |
| **Thyroid hormones** |  |  |  |  |  |  |
| Triiodothyronine | ng/dL | 963 | 10 | 20 | 993 |  |
| Thyroxine | ug/dL | 967 | 9 | 17 | 993 |  |
| Free thyroxine | ng/dL | 969 | 7 | 17 | 993 |  |
| Thyroid-stimulating hormone | uIU/mL | 928 | 15 | 50 | 993 |  |
| **Sex hormones** |  |  |  |  |  |  |
| Testosterone | ng/dL | 942 | 9 | 42 | 993 |  |
| Estradiol | pg/mL | 906 | 43 | 44 | 993 |  |
| Progesterone | ng/mL | 937 | 27 | 29 | 993 |  |
