# Supplementary material for: New trimester-specific reference intervals for clinical biochemical tests in Taiwanese pregnant women-cohort of TMICS
Source: PLoS One. 2020 Dec 14;15(12):e0243761. doi: 10.1371/journal.pone.0243761 (PMC7735596; doi:10.1371/journal.pone.0243761)
Supplement: S4 Table — (DOC) [file pone.0243761.s004.doc]

**S4 Table. 90% CI for the Reference intervals (RI) of pregnant women during their third trimester in Taiwan (TMICS).**

| **Item** | **Unit** | **n** |  | | | | **Mean ± SD** | **Median** | **IQR** |
| --- | --- | --- | --- | --- | --- | --- | --- | --- | --- |
|  |  |  | **90% CI for the RI percentile** | | | |  |  |  |
|  |  |  | **2.5^th^** | | **97.5^th^** | |  |  |  |
|  |  |  | **Lower Upper** | | **Lower Upper** | |  |  |  |
| **Hematology** |  |  |  |  |  |  |  |  |  |
| White blood cell | 10^3^/uL | 913 | 3.73 | 4.63 | 12.30 | 13.27 | 8.21 ± 2.32 | 8.20 | 3.10 |
| Red blood cell | 10^6^/uL | 913 | 3.20 | 3.30 | 4.51 | 4.63 | 3.91 ± 0.36 | 3.90 | 0.50 |
| Hemoglobin | g/dL | 924 | 9.50 | 9.90 | 13.30 | 13.80 | 11.49 ± 1.13 | 11.50 | 1.50 |
| Hematocrit | % | 913 | 29.90 | 30.60 | 41.20 | 42.50 | 36.15 ± 3.62 | 35.80 | 5.02 |
| Mean corpuscular volume | fL | 863 | 80.40 | 82.33 | 102.80 | 105.17 | 92.88 ± 6.39 | 92.70 | 8.50 |
| Mean corpuscular hemoglobin | pg | 886 | 24.27 | 26.00 | 32.80 | 33.40 | 29.63 ± 2.22 | 30.00 | 3.23 |
| Mean corpuscular hemoglobin concentration | g/dL | 919 | 27.56 | 29.40 | 34.30 | 34.70 | 31.68 ± 2.04 | 32.30 | 2.90 |
| Platelet | 10^3^/uL | 916 | 113.00 | 133.65 | 318.03 | 343.00 | 215.85 ± 57.3 | 213.50 | 80.00 |
| Neutrophil | % | 861 | 62.00 | 63.50 | 83.00 | 84.50 | 74.51 ± 5.78 | 74.90 | 8.30 |
| Lymphocyte | % | 867 | 10.06 | 11.40 | 27.07 | 29.80 | 18.55 ± 4.97 | 18.30 | 7.10 |
| Monocyte | % | 875 | 2.40 | 3.00 | 7.80 | 8.20 | 4.97 ± 1.44 | 4.90 | 1.90 |
| Eosinophil | % | 860 | 0.20 | 0.30 | 2.80 | 3.20 | 1.12 ± 0.72 | 0.95 | 1.10 |
| Basophil | % | 883 | 0.00 | 0.00 | 0.40 | 0.57 | 0.20 ± 0.13 | 0.20 | 0.20 |
| **Biochemical indicators** |  |  |  |  |  |  |  |  |  |
| Aspartate aminotransferase | U/L | 906 | 13.00 | 14.00 | 30.00 | 32.00 | 21.60 ± 5.09 | 21.00 | 8.00 |
| Alanine aminotransferase | U/L | 921 | 7.00 | 8.00 | 24.00 | 27.68 | 15.18 ± 4.60 | 15.00 | 7.00 |
| Creatinine | mg/dL | 931 | 0.36 | 0.40 | 0.67 | 0.72 | 0.53 ± 0.09 | 0.51 | 0.13 |
| Insulin | mIU/L | 898 | 2.80 | 3.63 | 79.24 | 95.57 | 25.62 ± 23.37 | 16.00 | 38.80 |
| Random blood sugar | mg/dL | 809 | 64.00 | 67.33 | 118.68 | 126.00 | 89.07 ± 16.02 | 86.00 | 23.00 |
| **Thyroid hormones** |  |  |  |  |  |  |  |  |  |
| Triiodothyronine | ng/dL | 963 | 80.00 | 87.33 | 178.00 | 194.00 | 131.10 ± 27.07 | 129.00 | 38.00 |
| Thyroxine | ug/dL | 967 | 7.50 | 8.40 | 13.90 | 14.64 | 10.99 ± 1.63 | 11.10 | 2.30 |
| Free thyroxine | ng/dL | 969 | 0.75 | 0.78 | 1.19 | 1.26 | 0.98 ± 0.12 | 0.98 | 0.16 |
| Thyroid-stimulating hormone | uIU/mL | 928 | 0.35 | 0.49 | 3.00 | 3.33 | 1.54 ± 0.83 | 1.38 | 1.17 |
| **Sex hormones** |  |  |  |  |  |  |  |  |  |
| Testosterone | ng/dL | 942 | 30.33 | 37.00 | 147.68 | 162.00 | 89.01 ± 36.06 | 81.00 | 55.00 |
| Estradiol | pg/mL | 906 | 6075.00 | 7612.46 | 25046.50 | 28430.70 | 15536.04 ± 5543.74 | 14571.80 | 7801.28 |
| Progesterone | ng/mL | 937 | 51.64 | 58.99 | 275.36 | 293.09 | 144.16 ± 66.15 | 129.10 | 95.12 |
