# Supplementary material for: New trimester-specific reference intervals for clinical biochemical tests in Taiwanese pregnant women-cohort of TMICS
Source: PLoS One. 2020 Dec 14;15(12):e0243761. doi: 10.1371/journal.pone.0243761 (PMC7735596; doi:10.1371/journal.pone.0243761)
Supplement: S5 Table — (DOC) [file pone.0243761.s005.doc]

| **S5 Table. Comparison of reference intervals between the first four weeks (weeks 28 to 31) and the last four weeks (weeks 37 to 40) of the third trimester of pregnant women.** | | | | | | | | | | | | |
| --- | --- | --- | --- | --- | --- | --- | --- | --- | --- | --- | --- | --- |
| **Item** | **Unit** | **Between 28 and 31** | | | |  | **Between 37 and 40** | | | | ***P value** | **False discovery rate** |
|  |  | **n** | **Median** | **2.5th** | **97.5th** |  | **n** | **Median** | **2.5th** | **97.5th** |  |  |
| **Hematology(excluded WBC,RBC,Hb,PLT)** |  |  |  |  |  |  |  |  |  |  |  |  |
| Hematocrit | % | 474 | 34.90 | 29.49 | 41.83 |  | 320 | 38.10 | 30.50 | 45.50 | < 0.001 | < 0.002 |
| Mean corpuscular volume | fL | 443 | 92.40 | 81.70 | 104.86 |  | 303 | 93.80 | 78.24 | 108.08 | 0.001 | 0.002 |
| Mean corpuscular hemoglobin | pg | 447 | 30.30 | 24.46 | 33.08 |  | 321 | 29.60 | 23.81 | 33.20 | < 0.001 | < 0.002 |
| Mean corpuscular hemoglobin concentration | g/dL | 474 | 32.60 | 27.99 | 34.70 |  | 326 | 30.90 | 26.24 | 34.20 | < 0.001 | < 0.002 |
| Neutrophil | % | 450 | 75.00 | 62.00 | 83.30 |  | 298 | 74.90 | 62.49 | 86.71 | 0.94 | 0.94 |
| Lymphocyte | % | 450 | 18.00 | 10.40 | 28.80 |  | 304 | 18.80 | 6.60 | 29.58 | 0.11 | 0.15 |
| Monocyte | % | 452 | 4.90 | 2.63 | 7.80 |  | 309 | 4.70 | 1.60 | 8.20 | 0.059 | 0.09 |
| Eosinophil | % | 437 | 1.00 | 0.30 | 3.10 |  | 314 | 0.80 | 0.10 | 2.71 | < 0.001 | < 0.002 |
| Basophil | % | 457 | 0.20 | 0.00 | 0.50 |  | 312 | 0.20 | 0.00 | 0.40 | 0.5 | 0.59 |
