# Supplementary material for: New trimester-specific reference intervals for clinical biochemical tests in Taiwanese pregnant women-cohort of TMICS
Source: PLoS One. 2020 Dec 14;15(12):e0243761. doi: 10.1371/journal.pone.0243761 (PMC7735596; doi:10.1371/journal.pone.0243761)
Supplement: S6 Table — (DOC) [file pone.0243761.s006.doc]

**S6 Table. The approval IRB numbers from National Health Research Institutes (NHRI) and other nine hospitals.**

| **Research sites** | **Hospital** | **IRB No.** |
| --- | --- | --- |
| Northern area | Taipei Veterans General Hospital | 2012-08-004AY |
|  | Cathay General Hospital | P101049 |
|  | Taipei City Hospital | TCHIRB-1030332 |
| Middle area | NHRI | EC1010501 |
|  | Hsinchu Cathay General Hospital | CGH-P101049 |
|  | Chung Shan Medical University Hospital | CS12082 |
|  | Changhua Christian Hospital | 120618 |
| Southern area | E-Da Hospital | EMRP35101N |
|  | Kaohsiung Municipal Siaogan Hospital | KMUHIRB- 2012-11-02(I) |
| Eastern area | Hualien Tzu Chi Hospital | ACT-IRB101-11 |
